# Methanogens acquire and bioaccumulate nickel during reductive dissolution of nickelian pyrite

**DOI:** 10.1128/aem.00991-23

**Published:** 2023-10-13

**Authors:** Rachel L. Spietz, Devon Payne, Eric S. Boyd

**Affiliations:** 1 Department of Microbiology and Cell Biology, Montana State University, Bozeman, Montana, USA; University of Michigan-Ann Arbor, Ann Arbor, Michigan, USA

**Keywords:** nickel famine, (NiFe)S_2_, great oxidation event, hydrogen, methane, hydrogenase, anaerobic, sulfide

## Abstract

Nickel (Ni) is a key component of the active site metallocofactors of numerous enzymes required for methanogenesis, including [NiFe]-hydrogenase, carbon monoxide dehydrogenase, and methyl CoM reductase, leading to a high demand for Ni among methanogens. However, methanogens often inhabit euxinic environments that favor the sequestration of nickel as metal-sulfide minerals, such as nickelian pyrite [(Ni,Fe)S_2_], that have low solubilities and that are not considered bioavailable. Recently, however, several different model methanogens (*Methanosarcina barkeri*, *Methanococcus voltae*, *Methanococcus maripaludis*) were shown to reductively dissolve pyrite (FeS_2_) and to utilize dissolution products to meet iron and sulfur biosynthetic demands. Here, using *M. barkeri* Fusaro, and laboratory-synthesized (Ni,Fe)S_2_ that was physically isolated from cells using dialysis membranes, we show that trace nickel (<20 nM) abiotically solubilized from the mineral can support methanogenesis and limited growth, roughly fivefold less than the minimum concentration known to support methanogenesis. Furthermore, when provided direct contact with (Ni,Fe)S_2_, *M. barkeri* promoted the reductive dissolution of (Ni,Fe)S_2_ and assimilated solubilized nickel, iron, and sulfur as its sole source of these elements. Cells that reductively dissolved (Ni,Fe)S_2_ bioaccumulated approximately fourfold more nickel than those grown with soluble nickel and sulfide but had similar metabolic coupling efficiencies. While the mechanism for Ni uptake in archaeal methanogens is not known, homologs of the bacterial Nik uptake system were shown to be ubiquitous across methanogen genomes. Collectively, these observations indicate that (Ni,Fe)S_2_ is bioavailable in anoxic environments and that methanogens can convert this mineral into nickel-, iron-, and sulfur-containing metalloenzymes to support methanogenesis and growth.

**IMPORTANCE:**

Nickel is an essential metal, and its availability has changed dramatically over Earth history due to shifts in the predominant type of volcanism in the late Archean that limited its availability and an increase in euxinic conditions in the early Proterozoic that favored its precipitation as nickel sulfide minerals. Observations presented herein indicate that the methanogen, *Methanosarcina barkeri*, can acquire nickel at low concentration (<20 nM) from soluble and mineral sources. Furthermore, *M. barkeri* was shown to actively reduce nickelian pyrite; use dissolution products to meet their iron, sulfur, and nickel demands; and bioaccumulate nickel. These data help to explain how *M. barkeri* (and possibly other methanogens and anaerobes) can acquire nickel in contemporary and past anoxic or euxinic environments.

## INTRODUCTION

Nickel is an essential metal, and its availability has changed dramatically over Earth history. Geochemical data indicate that, in the late Archean (~2.7 Gya), the mantel began to cool resulting in decreased eruption of Ni-bearing ultramafic rocks. Less emplacement of these rocks in near-surface environments decreased the exposure of their Ni-rich minerals to weathering, leading to an abrupt decline in Ni concentrations in oceans from ~400 nM at 2.7 Gya to ~200 nM at 2.5 Gya (1). At the same time, oxygen began accumulating (2, 3) which allowed for increased oxidative weathering of terrestrial sulfide minerals [e.g., pyrite (FeS_2_)], releasing sulfate (SO_4_^2-^) to oceans. This increased input of sulfate stimulated biological sulfate reduction [as reviewed in reference (4)] that likely led to sulfidic (i.e., euxinic) conditions, in particular near continental margins (5–7). Sulfide (HS^-^) readily reacts with thiophilic metals, including Ni, decreasing their soluble concentration due to the formation of metal sulfide clusters (e.g., NiHS^0^) and ultimately low solubility minerals [e.g., nickelian pyrite, (Ni,Fe)S_2_; millerite, NiS, and pentlandite, (Fe,Ni)_9_S_8_ (8–10)]. As such, expanded euxinic conditions due to the stimulation of sulfate reducers ~2.4 Gya likely further limited the availability of Ni. By 0.55 Gya and to the present, the concentration of Ni in oceans has been ~9 nM (11).

Methanogens have a high requirement for Ni (12, 13), as many of the key enzymes involved in methanogenesis and the Wood-Ljungdahl pathway of CO_2_ fixation have active sites or rely on cofactors that comprise this element. The systematic decreases in the availability of Ni over Earth’s history due to changes in volcanism and stimulation of sulfate reducers have been suggested to have caused a substantial contraction in the distribution and activity of methanogens during this time, which may have led to the rapid decline in atmospheric CH_4_ that, in turn, may have allowed for the rise in O_2_ ~2.4 Gya (1, 14). Central to evaluating the influence of Ni availability on the distribution and activity of methanogens is a more complete understanding of available Ni sources in methanogenic habitats and methanogen demand for Ni. A survey of five diverse methanogens grown with various methanogenesis substrates (i.e., H_2_/CO_2_, acetate, methanol, trimethylamine) showed Ni requirements for optimum growth ranging from 0.1 to 2 µM of added Ni (15). When Ni concentrations were provided at lower concentrations than these, impaired growth and reduced activity were observed (16). Importantly, however, the cultivation conditions in these studies included high concentrations of sulfide (HS^-^) as both a reducing agent and a sulfur source. As mentioned above, HS^-^ readily reacts with thiophilic metals, including Ni, which decreases soluble concentrations due to the formation of metal sulfide clusters and low solubility minerals.

To better constrain Ni requirements for methanogen growth and activity, it therefore becomes necessary to (i) identify the minimum concentration of Ni required for methanogenesis under non-sulfidic conditions and (ii) determine if Ni-sulfide minerals are bioavailable to methanogens. Several recent studies have shown that methanogens can reductively dissolve FeS_2_ (17–20) through what has been proposed to be a non-dedicated mechanism of extracellular electron transfer (EET) from the cell that requires direct contact with the mineral, unless exogenous electron transfer shuttles are used (17–19, 21). Furthermore, these studies of several methanogens (*Methanosarcina barkeri* Fusaro and MS, *Methanococcus voltae* A3, *Methanococcus maripaludis* S2) showed that Fe and S dissolution products meet the biosynthetic demands for these elements. This suggests the possibility that other thiophilic trace metals (e.g., Ni) that readily are sequestered by FeS_2_ could also be made available through reductive dissolution. Furthermore, experiments where a source of sulfur for cells other than HS^-^ (e.g., FeS_2_) would provide a mechanism to evaluate minimum Ni concentrations to support growth under non-sulfidic conditions.

In the present study, we determined the ability of the model methanogen, *M. barkeri* Fusaro to access Ni from (Ni,Fe)S_2_ and use the mineral as the sole source for Ni, Fe, and S for growth and methanogenesis. Related *Methanosarcina* species inhabit a diversity of habitats including marine sediments, the cow rumen, wastewater treatment facilities, and other freshwater environments (22), thus making *M. barkeri* Fusaro an ideal methanogen to examine the potential for (Ni,Fe)S_2_ to serve as a source of bioavailable Ni. Through (Ni,Fe)S_2_ leaching studies, we sought to examine the lower limit of Ni that supports methanogenesis and growth. Finally, we compared the Ni content of whole cells grown under nickel replete and deplete conditions provided with soluble or mineral [i.e., (Ni,Fe)S_2_] sources of Ni. The results are discussed as they relate to minimum requirements for Ni and mechanisms of its acquisition from minerals by methanogens in the euxinic environments they commonly inhabit on modern Earth and during the transition from the late Archean to the early Proterozoic.

## RESULTS AND DISCUSSION

### Synthesis, solubility, and reactivity of synthetic nanoparticulate (Ni,Fe)S_2_

**Fig 1:**
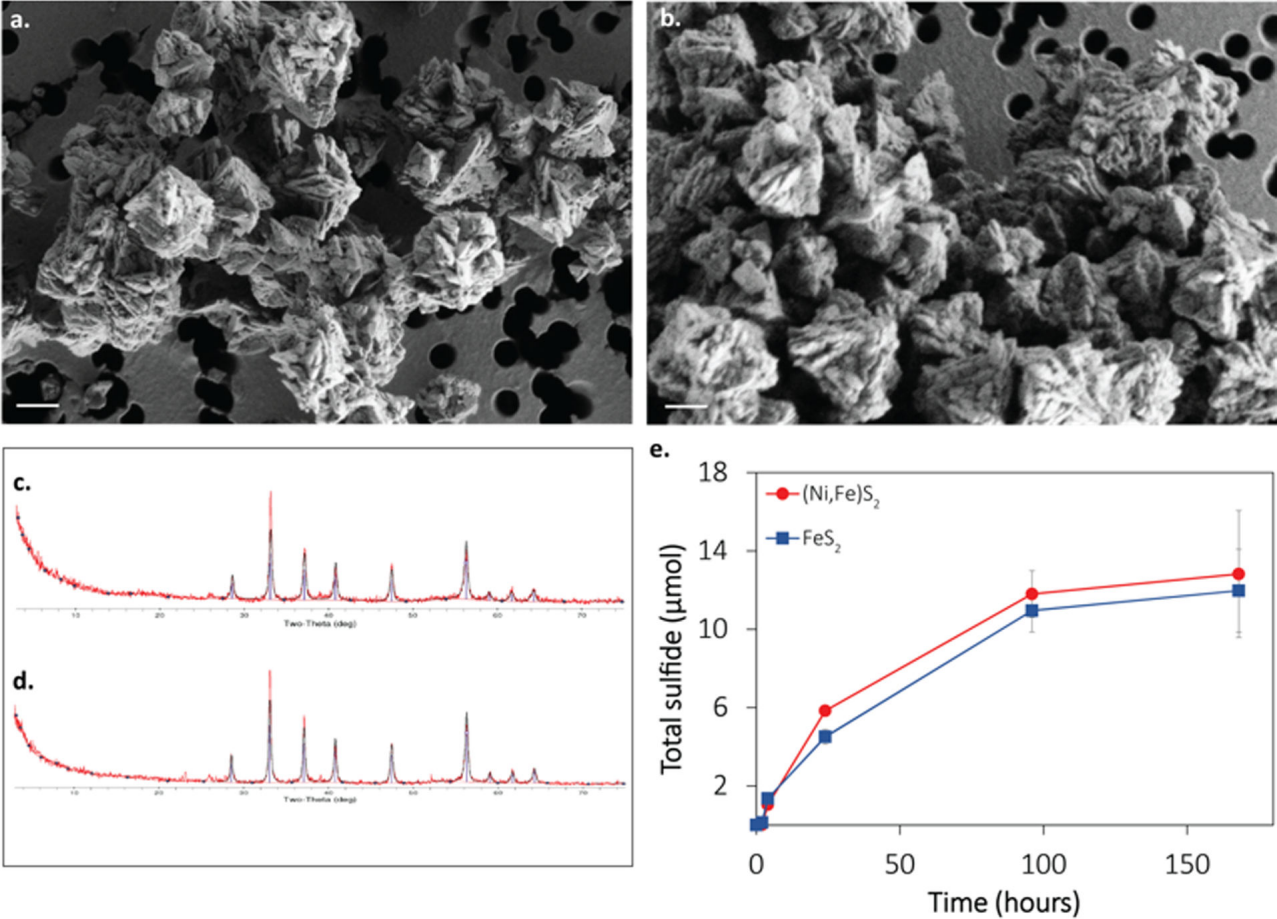
Synthetic nickelian pyrite (Ni,Fe)S_2_ has similar physical, chemical, and structural properties and reactivity as synthetic pyrite (FeS_2_). a-b, Field emission scanning electron micrographs of synthetic FeS_2_ framboids (**a**) and synthetic (Ni,Fe)S_2_ framboids (**b**) (scale bar = 250 nM in both panels). (**c and d),** X-ray diffraction (XRD) spectra for synthetic FeS_2_ and synthetic (Ni,Fe)S_2_. (**e)** The production of total sulfide (dissolved plus gas phase) from abiotic reduction of either synthetic FeS_2_ (blue squares and line) or (Ni,Fe)S_2_ (red circles and line) by H_2_ incubated at 38°C. No sulfide was detected in abiotic reactors containing FeS_2_ or (Ni,Fe)S_2_ when the headspace was N_2_ (data are not shown).

Synthetic (Ni,Fe)S_2_ was prepared following previously described methods for nanoparticulate FeS_2_ (17, 23) but with the replacement of 5 mol% Fe with Ni during the initial mackinawite synthesis step (see Materials and Methods for details). Briefly, the synthesis reaction proceeded through the formation of nickelian mackinawite [(Ni,Fe)S] by reacting Fe(II) and Ni(II) with stoichiometric HS^-^ followed by the polysulfide-dependent conversion of (Ni,Fe)S to (Ni,Fe)S_2_. Synthesized (Ni,Fe)S_2_ was washed several times with 1N HCl and once with 6N HCl to solubilize and thus remove surface-associated and/or unreacted metals. Synthesis of (Ni,Fe)S_2_ proceeded without any observed visual differences from the synthesis reaction for FeS_2_. The average size (~500 nM) and morphology (rhombohedral or framboidal) of (Ni,Fe)S_2_ grains were similar to FeS_2_ (Fig. 1a and b). The mineral composition of (Ni,Fe)S_2_, as assessed by X-ray diffraction spectroscopy, showed very minor spectral differences from FeS_2_ and thus was considered a 100% match (Fig. 1c and d). Using energy-dispersive X-ray spectroscopy (EDS), the weight % Ni, Fe, and S in synthetic FeS_2_ and (Ni,Fe)S_2_ were compared to predicted values based on the stoichiometry of reagents used in synthesis reactions. The wt % S in synthetic FeS_2_ was slightly lower than expected, and the wt % of Fe slightly higher (Table 1). This was also the case for (Ni,Fe)S_2_. However, the Ni content was comparable to the predicted wt % expected for (Ni,Fe)S_2_ and was below detection in FeS_2_, confirming Ni incorporation into the mineral matrix of the latter, similar to a previous report (24). Atomic absorption spectroscopy of fully acid-digested (Ni,Fe)S_2_ agreed with EDS measurements and indicated that 5.1 mol% of the Fe in the mineral was replaced by Ni (data not shown).

**TABLE 1:** The predicted and measured weight percent of sulfur, iron, and nickel in synthetic pyrite (FeS_2_) and nickelian pyrite ((Ni,Fe)S_2_)^*c*^

	Sulfur predicted (%)	Sulfur measured (%)^*a*^	Iron predicted (%)	Iron measured (%)^*a*^	Nickel predicted (%)	Nickel measured (%)^*a*^
FeS_2_	53.45	50.54 ± 0.67	46.55	49.46 ± 0.67	0.00	ND^*b*^
(Ni,Fe)S_2_	53.39	50.52 ± 0.63	44.17	46.18 ± 1.43	2.44	3.27 ± 1.15

^*a*^ Data represent the mean and standard deviation of the mean (*n* = 3).

^*b*^ ND, not detected.

^*c*^ Predicated elemental compositions were from the stoichiometry of reagents used in mineral syntheses, while measured elemental compositions were determined by energy-dispersive X-ray spectroscopy.

Bulk or specimen FeS_2_ is highly insoluble (9, 25), yet synthetic FeS_2_ with small grain size (~500 nM) and high surface area, such as synthesized herein, is likely to be more soluble (18). The same might also be expected for (Ni,Fe)S_2_. To examine anoxic dissolution of (Ni,Fe)S_2_, 2 mM of the mineral (as Fe) was incubated in 35 mL of anoxic ultrapure water for 48 h at 38°C under a N_2_ headspace. Following incubation, Ni and Fe were detected in the supernatant (0.22 µM filtered) at a concentration of 19 nM and 16 nM, respectively, despite Fe being at ~20-fold higher atom % abundance. This represents a solubilization of ~0.1% of the Ni and 0.001% of the Fe in the incubated (Ni,Fe)S_2_. Sulfide (HS^-^) was not detected following incubation (detection limit = 1.5 µM). Preferential release of Ni compared to Fe may be due to sorption of Ni on the surface of the mineral or diffusion from the mineral matrix, as has been described in other (Ni,Fe)S_2_ syntheses (24). Collectively, these data indicate that the synthetic (Ni,Fe)S_2_ used herein has a low solubility, and the concentrations of metals released into the solution are below the amounts commonly used to cultivate methanogen cells (0.1 to 2 µM), as discussed above and further below.

Previous studies have shown that synthetic FeS_2_ can be reductively dissolved by H_2_ at 38°C in aqueous solutions with pH 7.0, as evidenced by the detection of the dissolution product, HS^-^ (17, 18). To determine if the heterometal Ni influences the reactivity of (Ni,Fe)S_2_, 2 mM of synthetic (Ni,Fe)S_2_ or FeS_2_ was individually incubated in the presence of 200 µM dissolved H_2_ at 38°C under anoxic conditions, and the reaction progress was monitored by quantifying total sulfide (HS^-^ + H_2_S) (Fig. 1e). The rate and the maximum amount of total sulfide produced were not significantly different in reactors containing FeS_2_ or (Ni,Fe)S_2_, indicating that the reactivities of the two minerals were similar. Dissolved (< 0.22 µM) Fe or Ni was not detected in the supernatants of H_2_-reacted FeS_2_ or (Ni,Fe)S_2_ (data not shown). This is presumably attributable to Fe(II) and Ni(II) that were released during the dissolution process forming a complex with HS^-^ that is also released during mineral reduction (9, 10). Such complexes would likely eventually precipitate as FeS and NiS phases that are larger than the 0.22 µM pore size used to filter samples prior to metal determination.

### *M. barkeri* strain Fusaro can acquire Ni, Fe, and S from (Ni,Fe)S_2_

**Fig 2:**
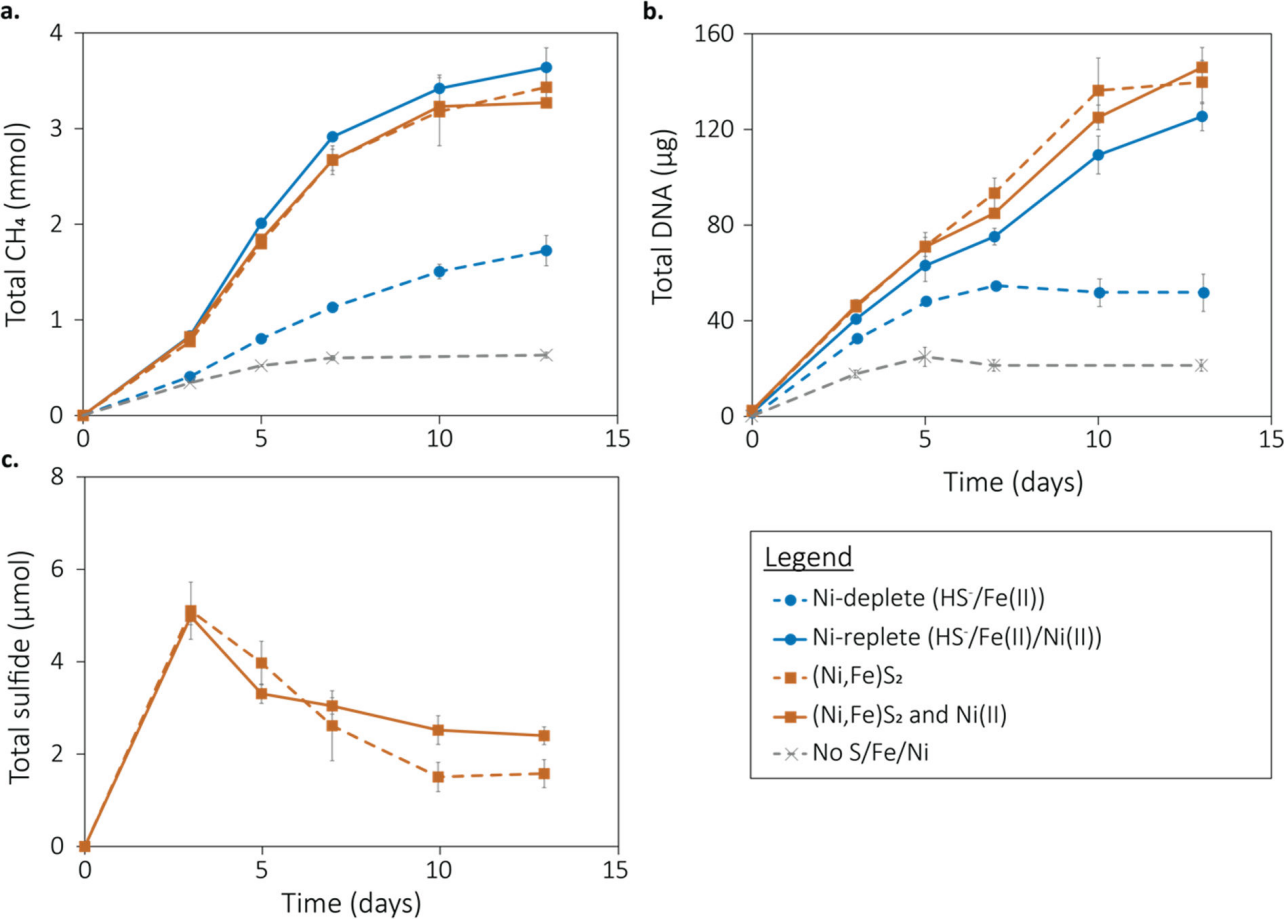
Growth and activity of *Methanosarcina barkeri* Fusaro with nickelian pyrite [(Ni,Fe)S_2_] or soluble sources of nickel (Ni), iron (Fe), and sulfur (S). Production of total methane (CH_4_) (**a**) and DNA (proxy for growth) (**b**) in cultures of *M. barkeri* Fusaro grown with acetate and methanol as methanogenesis substrates and with Fe(II) and HS^-^ with and without added Ni(II), as the sole source of Ni, Fe, and S or with (Ni,Fe)S_2_ with and without added Ni(II), as the source of Ni, Fe, and S, as specified in the legend. The production of total sulfide (dissolved plus gas phase) was only quantified in reactors provided with (Ni,Fe)S_2_ (c). CH_4_, DNA, or sulfide was not produced in abiotic controls (data not shown).

Several methanogen strains have been shown to reductively dissolve FeS_2_ to liberate Fe and S to support element demands for activity and growth (17, 18, 20, 21). Here, we tested whether *M. barkeri* Fusaro can also liberate Ni from (Ni,Fe)S_2_ during reductive dissolution to meet its high Ni demand (13, 16). Importantly, *M. barkeri* Fusaro was grown in low salt medium to limit contaminant metals in salts and to improve ICP-MS detection limits for Fe and Ni by avoiding unnecessary dilution. Furthermore, cells in this study were not grown with tryptone, yeast extract, or cysteine such as to limit the source(s) of Ni, Fe, and S to only those that were provided. This had the effect of decreasing the overall growth kinetics when compared to other studies where one or more of those substrates were provided (26, 27). *M. barkeri* Fusaro was first cultivated with soluble sources of Fe (20 µM Fe(II) added as FeCl_2_) and S (1 mM HS^-^ added as Na_2_S) under either Ni-replete (1 µM Ni(II) added as NiCl_2_) or Ni-deplete (no added Ni(II)) conditions. These were compared to cultures grown with no added Fe, S, or Ni to determine the effect of Ni limitation and combined Fe, S, Ni limitation on growth. Cells grown under Ni-deplete conditions produced 53% less CH_4_ and 67% less DNA (proxy for growth; 1.72 ± 0.16 mmol CH_4_, 39.80 ± 5.89 µg DNA, *n* = 3) after a 13-d incubation period compared to Ni-replete conditions (3.64 ± 0.20 mmol CH_4_, 120.31 ± 4.07 µg DNA, *n* = 3) (Fig. 2a and b), a significant decrease for both metrics (Student’s *t*-test: *p_CH4_ =* 1.02 × 10^−3^; *p_DNA_* = 2.96 × 10^−4^). When no Fe, S, or Ni was provided, cells produced 83% less CH_4_ and 95% less DNA (0.62 ± 0.03 mmol CH_4_, 6.30 ± 2.43 µg DNA, *n* = 3) than cells grown under Ni-replete conditions, also a significant decrease for both metrics (Student’s *t*-test: *p_CH4_* = 1.42 × 10^−5^; *p_DNA_* = 7.30 × 10^−4^). Together, these measures indicate that, like other methanogens (16), Ni limitation results in a growth defect in *M. barkeri* Fusaro.

*M. barkeri* Fusaro cells were then grown with (Ni,Fe)S_2_ as their sole Fe and S source with 1 µM added soluble Ni(II) or without added Ni. No significant difference in growth (CH_4_ or DNA production) was observed between these conditions (Fig. 2a and b). Furthermore, CH_4_ and DNA production in cultures provided with (Ni,Fe)S_2_ [either with added soluble Ni(II) or no added Ni] were not significantly different from those grown with soluble sources of Fe, S, and Ni (Fig. 2a and b). This indicates that Ni and Fe were not limiting during growth on (Ni,Fe)S_2_, either in the presence or absence of added soluble Ni(II). Yet, the cells under both conditions did not enter log phase growth, as previously seen in *M. barkeri* Fusaro growth curves (26, 27). Prior studies also typically provided *M. barkeri* Fusaro with cysteine as an additional sulfur source/reductant, suggesting the cells in this study might have been sulfur limited, a feature that may have been exacerbated by the cells growing as aggregates (discussed below). Limitation of sulfur solubilized from a mineral (in this case as an oxidant) has also been shown to keep the archaeon *Acidilobus sulfurireducens* from entering log-phase growth (28) and may point to enhanced turnover of cells during surface-dependent growth. Nonetheless, in this study the elimination of cysteine from the growth medium was necessary to demonstrate that (Ni,Fe)S_2_ can be used as a source of Ni, Fe, and S for *M. barkeri* Fusaro.

During growth on (Ni,Fe)S_2_, HS^-^ increased in concentration up to three days of incubation and then slowly declined (Fig. 2c) without a corresponding change in the pH of the growth medium pre- and post-growth (data not shown). The initial increase in total HS^-^ is indicative of biologically mediated (Ni,Fe)S_2_ reduction (17, 18, 21). The decrease in HS^-^ after three days of incubation may be due to the rate of HS^-^ production from mineral reduction being lower than the rate of HS^-^ assimilation since methanogens require more S than Fe and Ni for biosynthesis (13), and this would be the timeframe when the cells would typically enter log-phase growth. Alternatively, it is possible that the HS^-^ produced from mineral reduction complexed with metals in the growth medium and formed particles larger than 0.22 µM, the pore size used to filter the growth medium prior to HS^-^ determination.

**Fig 3:**
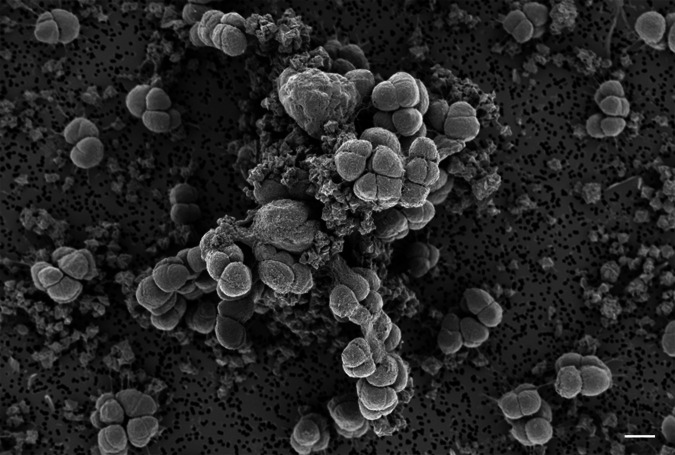
Field emission scanning electron microscopy image of *Methanosarcina barkeri* strain Fusaro in close association with (Ni,Fe)S_2_ rhombohedral nanoparticles during growth. *M. barkeri* Fusaro was grown with acetate and methanol as methanogenesis substrates and with (Ni,Fe)S_2_ as the sole source of nickel, iron, and sulfur. Scale bar equals 2 µM.

Previous studies have shown that, in the absence of soluble electron shuttles [e.g., hydrogen or anthraquinone-2,6-disulfonate (AQDS)], direct contact is required for methanogen-mediated FeS_2_ reduction (17, 18). Here, field emission scanning electron microscopy (FE-SEM) was used to visualize *M. barkeri* Fusaro cells provided with (Ni,Fe)S_2_ as their sole source of Ni, Fe, and S (Fig. 3). Cells grew as aggregates, common for this genus (22) when grown in low salt medium (22, 29), and appeared in close contact with the (Ni,Fe)S_2_ framboids. Thin filaments (likely dehydrated extracellular polymeric substance) were observed between cells and (Ni,Fe)S_2_ surfaces. The microscale association observed between *M. barkeri* Fusaro cells and (Ni,Fe)S_2_ supports macroscale observations that cells produce biofilms that encapsulate (Ni,Fe)S_2_ framboids, similar to what has been described for *Methanococcus voltae* and FeS_2_ [see Supplemental Videos in (17)]. This close contact is needed to support EET to reduce (Ni,Fe)S_2_ or FeS_2_ and liberate dissolution products required for biosynthesis (18). Further experiments using dialysis tubing to physically isolate cells from (Ni,Fe)S_2_ and its effects on growth are described below.

### *M. barkeri* Fusaro acquires low nM concentrations of Ni from abiotic dissolution of (Ni,Fe)S_2_

Experiments were conducted to determine if trace Ni released from abiotic dissolution of (Ni,Fe)S_2_ in growth medium (pH 7.0) at low temperature (38°C) could support methanogen activity and growth. Our previous experiments (discussed above) revealed that ~19 nM Ni was released from (Ni,Fe)S_2_ over 48 h of incubation at 38°C. To examine if this could support the growth of *M. barkeri* Fusaro, (Ni,Fe)S_2_ was sequestered in dialysis tubing with pore sizes equivalent to 12–14 kDa. By sequestering the mineral, direct contact between *M. barkeri* cells and (Ni,Fe)S_2_ was prohibited, thereby preventing the biological reduction of the mineral (18) and forcing cells to use only Ni leached from the mineral (Ni-leached). Production of CH_4_ and DNA was determined, since the cells grew as aggregates (see Materials and Methods) making it difficult to quantify growth using microscopy. CH_4_ and DNA production in these cultures was compared to (i) a negative control with no added Ni, Fe, or S; (ii) a positive control provided with 1 µM soluble Ni(II), 20 µM Fe(II), and 2 mM HS^-^ (Ni-replete); (iii) an experimental control with 20 µM soluble Fe(II) and 2 mM HS^-^ but no Ni (Ni-deplete); and (iv) an experimental control with 20 µM soluble Fe(II), 2 mM HS^-^, and 2 mM (as Fe) (Ni,Fe)S_2_ that was free in solution (Ni-mineral). Although dialysis membranes and clips underwent thorough washing prior to use in dialysis experiments (see Materials and Methods), untied membranes and clips were added to the negative control conditions to account for any trace addition of Fe and/or Ni to the growth medium from these materials.

**Fig 4:**
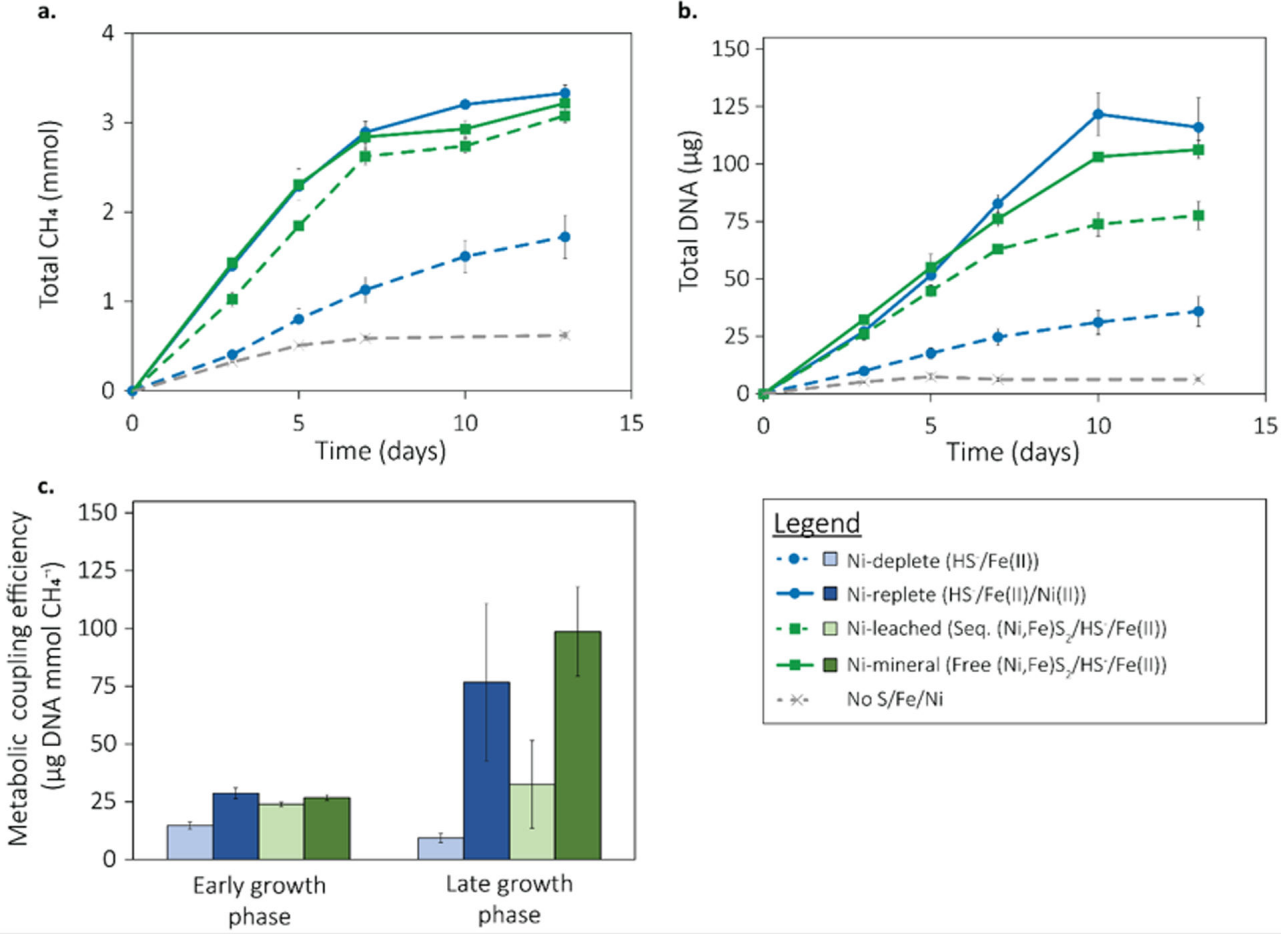
Access to nickelian pyrite [(Ni,Fe)S_2_] enhances reductive dissolution and increases growth efficiency in *Methanosarcina barkeri* Fusaro. Production of total methane (CH_4_) (**a**) and DNA (proxy for growth) (**b**) in cultures of *Methanosarcina barkeri* Fusaro grown with acetate and methanol as methanogenesis substrates and soluble forms of Fe, S, and Ni and/or nickelian pyrite [(Ni,Fe)S_2_] free in solution (Free) or sequestered in dialysis tubing (Seq.), as specified in the legend. (**c**) The metabolic coupling efficiency (i.e., µg of DNA produced per mmol of CH_4_ produced) calculated during the early phase (0–7 days) and during the late phase (7–13 days) of growth for each condition. CH_4_, DNA, or sulfide were not detected in abiotic controls (data not shown).

Ni-deplete grown cells provided with soluble Fe(II) and HS^-^ produced 48.3% less CH_4_ and 69.0% less DNA (1.72 ± 0.24 mmol CH_4_, 35.88 ± 6.55 µg DNA, *n* = 4) than Ni-replete grown cells (3.33 ± 0.09 mmol CH_4_, 122.00 ± 12.93 µg DNA, *n* = 4) (Fig. 4a and b), a significant decrease (Student’s *t*-test: *p_CH4_* = 1.42 × 10^−4^; *p_DNA_* = 7.56 × 10^−4^) similar to the observations presented in Fig. 2. Ni-replete-grown cells produced CH_4_ and DNA at levels similar to Ni-mineral-grown cells. However, while Ni-leached-grown cells produced CH_4_ that was comparable to the Ni-replete-grown cells (Fig. 4a), they produced significantly less DNA than both Ni-replete- (Student’s t-test: *p_DNA_* = 7.18 × 10^−3^) and Ni-mineral-grown cells (*p_DNA_* = 3.55 × 10^−3^) (Fig. 4b). This indicates that Ni leached from (Ni,Fe)S_2_ alone can support methanogenesis by *M. barkeri* Fusaro but growth at such low Ni concentrations is suboptimal.

To further explore the effect of Ni, Fe, and S sources on the growth of *M. barkeri* Fusaro, the metabolic coupling efficiency, or the ability of cells to couple their energy metabolism to production of biomass (measured here as µg DNA/mmol CH_4_), of cells cultivated under the varying conditions was calculated during the early (days 0–7) and late phases (days 7–13 ) of growth. Prior to this determination, however, a series of experiments was performed that showed that FeS_2_ has no effect on the extraction and recovery of *M. barkeri* DNA (Fig. S1). During the early phase of growth (days 0–7), metabolic coupling efficiencies were similar across the three conditions where Ni was supplied either as soluble Ni (as NiCl_2_; Ni-replete) or mineral Ni (as (Ni,Fe)S_2_) that was sequestered (Ni-leached) or free in solution (Ni-mineral) (Fig. 4c). In contrast, cells grown with soluble Fe(II) and HS^-^ but with no Ni (Ni-deplete) showed a decreased metabolic coupling efficiency during the early phase growth, which is attributed to Ni limitation that developed during this growth period.

During the late-phase growth (days 7–13) metabolic coupling efficiencies further diverged from those during the early phase growth but in unexpected ways. In the case of the Ni-replete- and Ni-mineral-grown cells, metabolic coupling efficiencies at late phase were threefold and fourfold higher than in early phase, respectively. In contrast, metabolic coupling efficiencies of the late-phase Ni-deplete- or Ni-leached-grown cells were not significantly different from cultures at early phase. It is not immediately clear why metabolic coupling efficiencies varied as they did in early phase versus late-phase cultures but it is unlikely to be attributable to minerals influencing DNA extraction or recovery efficiencies (Fig. S1). Comparing across treatments, Ni-replete cells had significantly higher metabolic coupling efficiencies than the Ni-deplete cells (Student’s *t*-test, *P* = 8.58 × 10^−3^, *n* = 3), while the Ni-mineral grown cells had significantly higher metabolic coupling efficiencies than Ni-leached cells (*P* = 9.29 × 10^−3^, *n* = 3). Collectively, these results demonstrate that cells can acquire additional Ni from (Ni,Fe)S_2_ through reductive dissolution, and this supports the growth that is comparable to those grown under Ni-replete conditions.

### Bioaccumulation of Ni from (Ni,Fe)S_2_ during reductive dissolution

The amount of Ni bioaccumulated in *M. barkeri* Fusaro biomass in Ni-replete and Ni-deplete growth conditions with either soluble or mineral sources of Ni was determined. However, because *M. barkeri* Fusaro directly attaches to (Ni,Fe)S_2_ during growth, it was first necessary to develop a cultivation system whereby reductive dissolution could be driven by cells that were physically isolated from the bulk mineral to avoid mineral contamination of biomass. We have shown that the synthetic electron shuttle, AQDS, facilitates EET from *M. barkeri* to FeS_2_ and allows for growth when the mineral is sequestered in dialysis tubing (18). Thus, growth experiments were conducted to determine the Ni content of cells provided with AQDS and with Fe(II), HS^-^, and (Ni,Fe)S_2_ sequestered in dialysis tubing (Ni-mineral), and these were compared to (i) Ni-replete cells grown with soluble Fe(II), HS^-^, and Ni(II); (ii) Ni-deplete cells grown with soluble Fe(II), HS^-^, and no Ni; and (iii) Ni-leached cells grown with Fe(II), HS^-^, and (Ni,Fe)S_2_ sequestered in dialysis tubing without added AQDS. Again, control cultures contained untied dialysis tubing and clips to control for contaminant elements that may contribute to the growth medium. The concentration of total dissolved (0.22 µm filtered) Ni in each cultivation reactor was determined pre- (0 days) and post-cultivation (13 days) for these conditions to attempt to account for the Ni present in each. Furthermore, prior to elemental analysis, cells were washed with nitriloacetic acid (NTA) to minimize the contribution of cell surface-associated Ni. The concentration of NTA used herein (5 mM) to remove sorbed metals from cells is less than that (10 mM) has been previously used to remove sorbed metals from the methanogen, *Methanocaldococcus jannaschii*, while maintaining cell viability (30). That said, bioaccumulation is defined for the purposes of this study as cell-associated since it is not definitively known if the metal was associated with extracellular components (sorption) or was intracellular.

**Fig 5:**
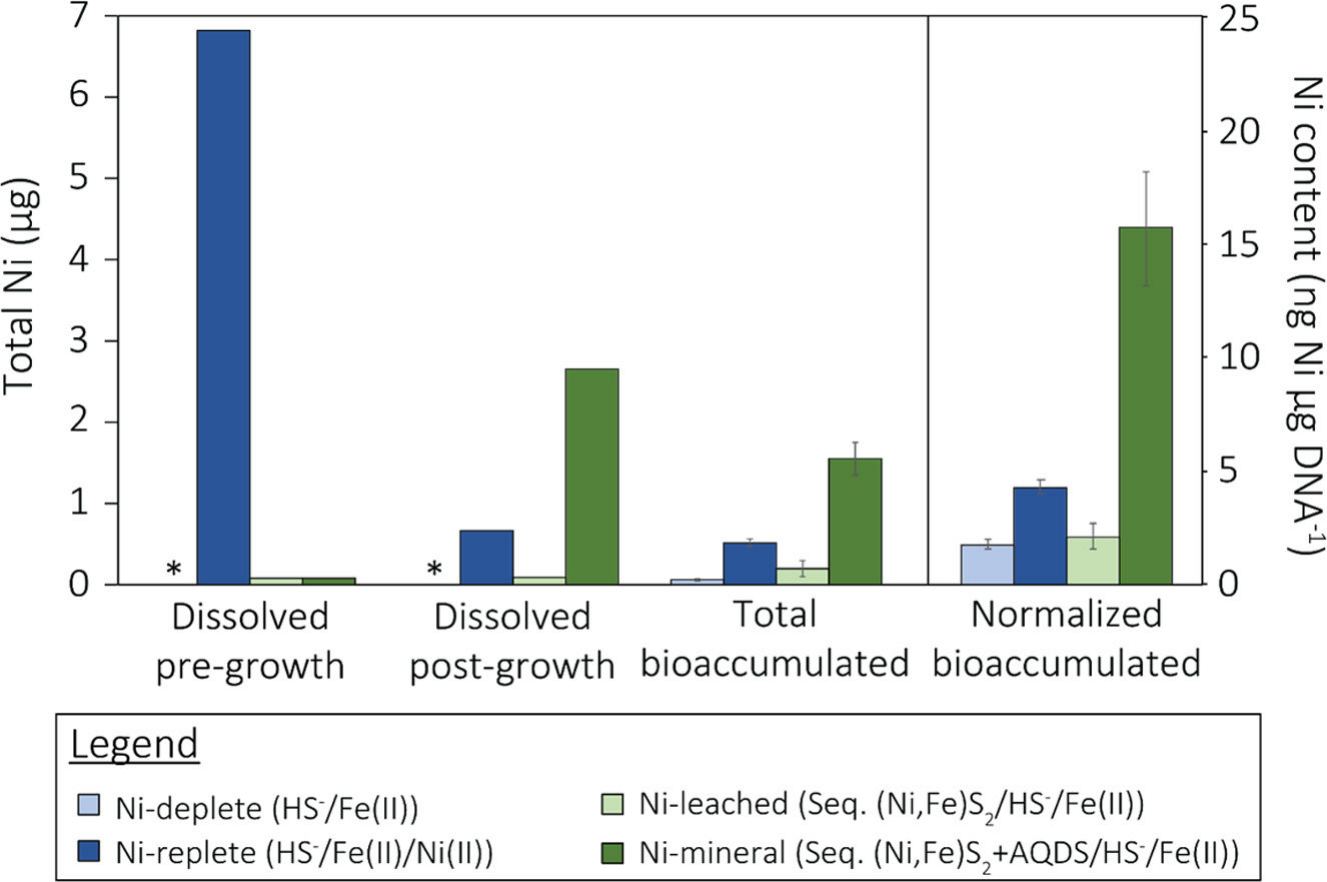
*Methanosarcina barkeri* Fusaro bioaccumulates nickel released through reductive dissolution of nickelian pyrite [(Ni,Fe)S_2_]. Total dissolved (< 0.22 µM) nickel (Ni) quantified in each reactor before methanogen growth (day 0, pre-growth) or following methanogen growth (day 13, post-growth) and the total bioaccumulated Ni in *M. barkeri* Fusaro biomass. Cells were grown with acetate and methanol as methanogenesis substrates and soluble forms of Fe, S, and Ni and/or (Ni,Fe)S_2_ sequestered in dialysis tubing (Seq.) with or without a synthetic electron shuttle, AQDS, provided to facilitate reductive dissolution of the mineral, as specified in the legend. Normalized bioaccumulated Ni content was calculated by dividing the total bioaccumulated Ni content in the biomass for each reactor by the total amount of DNA that was recovered from the biomass of each reactor. *, not detected.

The total dissolved Ni in each cultivation condition (75 mL culture volume in 165 mL serum bottle) varied pre- and post-growth, indicating differences in availability over time (Fig. 5). The Ni content in the medium of Ni-replete [1 µM Ni(II) provided initially] pre- and post-culture growth was 6.82 µg (1.54 µM, *n* = 1) and 0.66 µg (0.150 µM, *n* = 1), respectively. The decrease in Ni cannot be attributed entirely to assimilation as the total bioaccumulated Ni content of harvested biomass in each culture averaged 0.51 ± 0.05 µg (*n* = 4), indicating a substantial fraction (~5.7 µg) of Ni was precipitated by HS^-^ as Ni-sulfide phases that were larger than 0.22 µM (pore size of filters used). In contrast, the Ni-deplete medium had no detectable Ni (lower limit of detection, <0.04 µg Ni or <9 nM) pre- or post-growth, and the total Ni accumulated was ~0.06 ± 0.01 µg (*n* = 4), a factor of ~8 less than Ni-replete grown cells. In cultures provided with AQDS and sequestered (Ni,Fe)S_2_ (i.e., Ni-mineral), the amount of dissolved Ni in the medium increased from 0.08 µg (18 nM, *n* = 1) preincubation to 2.65 µg (0.602 µM, *n* = 1) post-incubation, a nearly 30-fold increase. In contrast, the Ni content of the medium from cultures provided with sequestered (Ni,Fe)S_2_ but without AQDS (i.e., Ni-leached) remained nearly the same before (0.08 µg or 18 nM, *n* = 1) and after 13 d of growth (0.09 µg or 20 nM, *n* = 1). Furthermore, the amount of Ni bioaccumulated in Ni-mineral cultures was ~10-fold higher than Ni-leached cultures (Ni-mineral: 1.55 ± 0.2 µg; Ni-leached: 0.2 ± 0.09 µg, *n* = 4).

Normalization of the amount of Ni bioaccumulated to DNA (proxy for growth) only further pronounced the differences in Ni availability and accumulation in the various culture conditions, with the highest normalized bioaccumulated Ni content in Ni-mineral cells (Fig. 5). The Ni content of Ni-mineral grown cells was approximately fourfold higher than Ni-replete-grown cells (Ni-mineral: 15.70 ± 2.5 ng Ni/µg DNA; Ni-replete: 4.22 ± 0.33 ng Ni/µg DNA, *n* = 4), and the difference was significant (Student’s *t*-test, *P* = 9.61 × 10^−6^, *n* = 4). Importantly, the Ni content of *M. barkeri* Fusaro grown in batch or continuous culture and with different carbon sources and methanogenesis substrates (i.e., methanol, acetate, H_2_/CO_2_, trimethylamine) with HS^-^ as sulfur source/reducing agent was previously shown to vary by up to a factor of two (13). This suggests that broad changes in carbon and methanogenesis pathways are unlikely to solely account for the Ni content of cultures observed herein. Furthermore, the Ni content of dried biomass from Ni-replete, Ni-deplete, Ni-mineral, and Ni-leached cultures was 35 ± 5.0, 6.4 ± 1.0, 163 ± 12.8, and 10 ± 2.0 ppm, respectively (Table S1). Values for Ni-deplete, Ni-leached, and Ni-replete cultures were twofold to 10-fold lower than what has been measured previously (range of 60–150 ppm) for a variety of methanogens grown on various substrates and with HS^-^ as sulfur source (13). Only Ni-mineral-grown cells fall within this previously determined range (Table S1). As mentioned above, cells in this study were washed with NTA (5 mM) and then with MQ water to dissociate and remove membrane-associated metals or metal clusters prior to element analysis. In contrast, previous reports of Ni content per cell were from methanogen cultures that were washed with only water prior to analysis (13), which may have led to an overestimation of the Ni content of methanogen biomass due to sorption of Ni or Ni sulfide clusters. Again, the concentration of NTA used in this study was half of that used previously and which was shown to not affect viability and thus membrane integrity in *M. jannaschii* (30).

### Probing potential mechanisms of Ni uptake in methanogens

Little is known of nickel uptake in methanogens; however, in other cells, it must be modulated to avoid metal toxicity (31, 32), and the same is likely true for methanogens. In *E. coli*, the NikR repressor binds to the promoter region of the *nikABCDE* operon when Ni is present, preventing transcription (33, 34). A prior bioinformatics study of NikR binding sites in eight methanogen genomes identified homologs of a second putative ABC transporter involved in cobalt (Co) uptake (CbiMNQO) near genomic regions encoding Ni-dependent enzymes (i.e., [NiFe]-hydrogenase, CODH) (35). Due to their location near genes encoding Ni-dependent enzymes in methanogen genomes, these homologs were hypothesized to be involved in maintaining Ni homeostasis and have been biochemically shown to have preference for uptake of Ni in several bacteria. As such, the genes were designated *nikMNQO* to differentiate them from Co uptake systems (35). NikQ and NikM are thought to be permeases, NikN has no predicted function, and NikO is predicted to be an ATPase that together functions as a high-affinity Ni^2+^ transport system (<100 nM) regulated by NikR (35).

Using a previously compiled database containing 301 methanogen genomes from the Department of Energy-Joint Genome Institute (DOE-JGI) (36), the distribution of genes encoding NikMNQOR homologs was examined (Table 2; Table S2). Every genome analyzed had at least one homolog of a component of the Nik system, with every genome encoding a homolog of NikO (ATPase) and oftentimes multiple homologs, which likely also included Co transport paralogs. Nearly all (98.0%) genomes encoded homologs of NikR. Most genomes defined as being complete (empirically defined as those with >95% estimated completeness through CheckM analyses as reported by DOE-JGI for each genome) encoded copies of both NifQ and NikM, with those that did not typically encode at least one homolog of NikQ or NikM. The genomes that did not encode a NikQ or NikM homolog were from an organism belonging to the more recently diverging order Methanocellales (*Methanocella paludicola* SANAE) and three organisms belonging to the early diverging order Methanopyrales (*Methanopyrus kandleri* AV19, *Methanopyrus* KOL6, and *Methanopyrus* SNP6). Together, these results suggest that the Nik system may be involved in Ni homeostasis in diverse methanogens, including those that are more early evolved [termed Type I based on phylogenetic branching (37, 38)] and those that are more recently evolved (termed Type II).

**TABLE 2:** Distribution of genes encoding homologs of NikMNQOR among 301 methanogen genomes

Taxonomic group			NikM	NikN	NikQ	NikO	NikR
	Genomes	Complete^*a*^	ATP-binding protein	Permease protein	Unknown	Permease protein	Transcriptional regulator
**Type I**							
Methanobacteriales	*102*	*78*	102	98	95	102	99
Methanococcales	*20*	*19*	20	18	19	20	20
Methanopyrales	*3*	*3*	0	0	3	0	3
**Type II**							
Methanocellales	*4*	*4*	3	1	0	3	4
Methanomicrobiales	*25*	*20*	23	19	17	25	23
Methanosarcinales	*131*	*122*	131	123	124	131	131
**Unclassified**							
Bathyarchaeota	*3*	*0*	1	0	0	3	3
Methanomassiliicoccales	*10*	*6*	6	7	0	10	10
Methanonatronarchaeales	*1*	*1*	0	0	0	1	1
Verstraetearchaeota	*2*	*1*	0	0	0	2	0

^*a*^ Finished defined as having >95% estimated completeness by CheckM.

Potentially in support of a role in Ni uptake, two gene clusters that encode homologs of Nik/CbiMNQO were identified in the Type I methanogen *Methanococcus maripaludis* S2 (locus tags MMP1481-1484 and MMP0885-MMP0889), one of which (MMP1481-1484) is co-localized with genes encoding the membrane-associated [NiFe]-hydrogenase, Eha (locus tags MMP1448-1467). A previous study generated transposon insertions randomly in the genome of *M. maripaludis* S2 and used these to identify the essentiality of encoded RNAs and proteins during growth with H_2_/CO_2_ (39). Scores of <3 and <2 were deemed diagnostic of essential protein-encoding genes in rich and minimal medium, respectively, and scores of >11 and >5 were deemed diagnostic of possibly nonessential protein-encoding genes in rich and mineral medium. In rich medium containing yeast extract, casamino acids, acetate, vitamins, cysteine, and coenzyme M, the essentiality of MMP1481-1484 ranged from 18 to 48 and MMP0885-MMP0889 ranged from 9 to 46 indicating that they are potentially nonessential under this growth condition. However, in mineral medium with cysteine as the only organic amendment, the essentiality of MMP1481-1484 ranged from 6 to 14 and MMP0885-MMP0889 ranged from 0 to 18; the essentiality of Eha (MMP1448-1467) minus subunits PQRS (MMP1463-1466) was <3 regardless of conditions. This is consistent with a potential role for MMP1481-1484 in Ni acquisition and homeostasis in *M. maripaludis* S2. The essentiality scores (borderline nonessential) may also suggest alternative mechanisms of acquiring Ni in *M. maripaludis* S2 and possibly other methanogen strains such as *M. barkeri* Fusaro or some level of redundancy with other uptake systems.

While Nik may be involved in maintaining Ni homeostasis in methanogen cells, it does not necessarily help to explain bioaccumulation of Ni in those cells, if the Ni is intracellular. The Nik system is specific for Ni^2+^ (32), which, like other thiophilic metals (e.g., Fe^2+^), is likely to be present as a nickel-sulfide in sulfidic medium or environments (8–10). It is possible that the high content of Ni is due to sorption on the cell surface, although steps were taken to minimize this possibility (i.e., wash with NTA). An alternative explanation for Ni bioaccumulation in cells grown with (Ni,Fe)S_2_ sequestered in the presence of AQDS in this study comes from experiments conducted with the methanogen *Methanococcus voltae* when grown on FeS_2_ (19). In that study, *M. voltae* cells grown with FeS_2_ as the sole source of Fe and S exhibited approximately twofold higher Fe contents than cells grown with FeCl_2_ and HS^-^. The excess Fe was shown to be stored as an intracellular thioferrate-like mineral (19). Despite FeS_2_-grown *M. voltae* cells apparently being replete with Fe, shotgun proteomics of these cells revealed up-expression of the Fe(II) transporter (FeoB) and the Fe(II) regulators FeoA and DtxR relative to FeCl_2_/HS^-^-grown cells. In bacteria, the Feo system is upregulated in response to Fe(II) limitation and is involved in Fe(II) transport (40). In methanogens, homologs of genes encoding FeoAB system are ubiquitous (36), and in *M. maripaludis* S2, genes encoding FeoAB are essential (39). In the case of up-expression of FeoAB and DtxR in *M. voltae* cells grown with FeS_2_, it was posited that cells incorrectly sensed Fe limitation during growth on FeS_2_ because Fe(II) was complexed with HS^-^ (17). It was suggested that passive transport of uncharged iron-sulfur aqueous clusters resulted in the bioaccumulation of Fe. Like Fe(II), Ni(II) has a strong affinity for HS^-^, although it is not clear if charged [(Ni(HS)_2_)^0^] or neutral [Ni(HS)^+^] aqueous clusters are predominant under the conditions of the experiments conducted herein (9). If the Ni-sulfide clusters are uncharged, it may lead to intracellular bioaccumulation of Ni in *M. barkeri* Fusaro in a manner similar to that previously observed with Fe in *M. voltae*. Additional work is warranted to determine (i) the mechanism of Ni uptake/transport/bioaccumulation in these cells, (ii) the form of Ni that is bioaccumulated, either through extracellular sorption or as intracellular phases, and (iii) the extent that Ni from mineral sources such as (Ni,Fe)S_2_ supports methanogens (and potentially other anaerobes) in natural environments. Regardless, the observations presented herein help to explain how methanogens acquire Ni at low concentrations and from mineral sources that are expected to be predominant in sulfidic habitats, both today and in the geologic past.

## MATERIALS AND METHODS

### Preparation of synthetic and natural minerals

Synthetic nickelian pyrite (Ni,Fe)S_2_ was prepared by modifying previously described FeS_2_ synthesis methods (17, 23) to include a Ni impurity at a final concentration of 1.67%. Specifically, two separate 50 mL solutions (A and B) were prepared in sterile, anoxic ultrapure MilliQ water (MQ H_2_O) inside of an anaerobic chamber (97.5%:2.5% N_2_:H_2_). Solution A contained 57 mmol FeSO_4_·7H_2_O and 3 mmol NiCl_2_·6H_2_O, while solution B contained 60 mmol Na_2_S. Solutions A and B were combined and mixed on a stir plate with a magnetic stir bar for 10 mins. Next, 2.1 g of elemental sulfur (S^0^; previously baked at 95°C to dry and sterilize) was added to the mixture of solutions A and B, and it was stirred for an additional 15 min. The mixture was then transferred to a glass serum bottle, sealed with a blue butyl rubber stopper, and crimped closed. The bottle was removed from the anaerobic chamber, and the solution was bubbled for 1 h/L with sterile N_2_ gas that had been passed over a heated (210°C), H_2_-reduced copper column. All chemicals used in the synthesis were of American Chemical Society grade, and all glassware was washed with 10% nitric acid prior to use. The synthesis reaction was then allowed to incubate in the dark in the sealed anaerobic bottle for four days at 65°C, followed by an additional four days at 85°C. After the synthesis had completed, to remove any unreacted HS^-^, Fe(II), FeS, Ni(II), NiS, and S^0^, the synthesized (Ni,Fe)S_2_ was washed via centrifugation and decanting initially with 1N HCl, then with boiling 6N HCl, then twice with MQ H_2_O, then three times with >99.5% acetone, and finally five times with sterile, anoxic MQ H_2_O within an anaerobic chamber. The final synthetic (Ni,Fe)S_2_ slurry was transferred to a sterile, anoxic, acid-washed serum bottle and then capped and sealed prior to removal from the anaerobic chamber and sparging with sterile and anoxic N_2_ (see above) to remove residual H_2_.

### Mineral characterization and reactivity

Washed minerals were dried under a stream of 0.22 µM filtered ultrahigh purity N_2_ gas prior to characterization on a SCINTAG X-1 system X-ray powder diffraction (XRD) spectrometer (XRD; Eigenmann GmbH, Mannheim, Germany) and a Zeiss SUPRA 55VP field emission scanning electron microscope (FE-SEM; Zeiss, Oberkochen, Germany) equipped with an energy dispersive X-ray spectrometer (EDS). A small aliquot of the mineral sample (~0.1 g) was dried under a stream of 0.22 µM filtered ultrahigh purity N_2_ gas and then dissolved in 40% trace metal-free HNO_3_ for three days at room temperature (~21°C). The dissolved mineral was diluted with MQ H_2_O to 3% HNO_3_ and passed through a 0.22 µM PTFE syringe filter prior to quantification of Fe and Ni *via* atomic absorption (AA) spectroscopy on an Agilent 240 FS instrument (Agilent Technologies Inc., Santa Clara, CA) equipped with Fe and Ni lamps utilizing an acetylene/air mixture (11:60 lb/in^2^ partial pressures) as a fuel source. The Fe and Ni contents for the samples were determined using standard curves prepared from 1,000 ppm Fe or Ni standards (Ricca Chemical Company, Arlington, TX), respectively. All dilutions and standards were prepared in fresh 3% HNO_3_.

To compare the reactivity of synthetic FeS_2_ to that of (Ni,Fe)S_2_, minerals were reacted with H_2_, and HS^-^, a product of FeS_2_ reduction, was quantified following previously described methods (18). Briefly, 2 mM (as Fe) of FeS_2_ or (Ni,Fe)S_2_ was incubated in 35 mL of anoxic MQ H_2_O with either 100% N_2_ headspace (negative control) or 90%:10% N_2_:H_2_ headspace (experimental control). Triplicate reactors for each headspace condition and each mineral type were incubated for seven days at 38°C. Dissolved HS^-^ was measured at 0, 2, 24, 96, and 168 h by removing liquid samples with a N_2_-purged needle and syringe, passing the sample through a 0.22 µM filter, and quantifying HS^-^ via the methylene blue assay (41). Dissolved HS^-^ concentrations were converted to total sulfide (HS^-^ + H_2_S) using Henry’s law. At the end of incubation, the supernatant from each reactor was filtered through a 0.22 µM PTFE syringe filter and then acidified in 5% trace metal-free HNO_3_ prior to the quantification of dissolved Fe and Ni *via* inductively coupled plasma mass spectrometry (ICP-MS) on a Thermo iCAP Q ICP-MS (Thermo Fisher Scientific, San Jose, CA) at the Montana Bureau of Mines and Geology Analytical Laboratory at Montana Technological University.

### Cultivation conditions

*M. barkeri* strain Fusaro was purchased from the American Type Culture Collection (ATCC-BAA-2329). Although it has been shown that *M. barkeri* Fusaro disaggregates in high salinity medium, the high salt content would have prohibited the accurate quantification of Fe and Ni on ICP-MS and AA spectroscopy. Thus, the cells were grown in low salinity medium as previously described (18). All growth medium and amendments were prepared without Fe, Ni, or S in ultrapure, MQ water and using acid (10% HNO_3_)-washed glassware. The base salts medium contained (in g/L): NaCl, 1.00; MgCl_2_ ⋅ 6H_2_O, 0.40; NH_4_Cl, 0.50; KCl, 0.50; CaCl_2_ ⋅ 2H_2_O, 0.10; KH_2_PO_4_, 0.15; NaHCO_3_, 2.0. Prior to adding the NaHCO_3_, the remaining base salts medium was boiled for 10 min, then purged with anoxic N_2_ gas (see above) for 1 h/L. The sparged medium was capped, sealed, and then moved into an anaerobic chamber where the NaHCO_3_ was added, and the pH was adjusted to 7.0 using 1N HCl. The medium (75 mL) was dispensed into 165 mL serum bottles, capped with a blue butyl rubber stopper, and sealed with crimp caps before removing from the anaerobic chamber. The headspace of bottles was exchanged with N_2_:CO_2_ (80%:20%) gas for 15 min, before autoclaving.

Prior to inoculation, the medium was amended with 1% (vol/vol) Wolfe’s vitamins, 1% SL-10 trace metals (no Fe- or Ni-containing components), 0.5% (vol/vol) or 123 mM methanol, and 4 mM acetate. All cultures were grown with a N_2_:CO_2_ (80%:20%) headspace pressurized to 1.72 atm. Where indicated, Fe(II) (as FeCl_2_) was added to a final concentration of 20 µM, Ni(II) (as NiCl2) was added to a final concentration of 1 µM, sulfide (as Na_2_S) was added to a final concentration of 2 mM, FeS_2_ [as Fe(II)] was added to a final concentration of 2 mM, and (Ni,Fe)S_2_ was added to a final concentration of 2 mM. Cultures were inoculated with a 5% (vol/vol) transfer of cells from a mid-log phase culture that was first pelleted via centrifugation (4,696 × *g* for 20 min at 4°C) and then washed with 5 mM nitriloacetic acid in sterile and anoxic medium; the nitrilotriacetic acid (NTA) was used as a chelator to remove residual metals, including Fe and Ni.

For experiments where (Ni,Fe)S_2_ was sequestered to prohibit direct physical contact between the mineral and cells, 12–14 kDa dialysis tubing and clips (Spectrum Laboratories, Rancho Dominguez, CA) were first washed in MQ water and sterilized in ethanol as previously described (17). Once washed and sterilized, one end of the tubing was clipped and then the tubing was filled with a (Ni,Fe)S_2_ slurry and sealed with the other clip. Sealed tubing containing (Ni,Fe)S_2_ was added to the culture bottles, and the bottles were capped and sealed within the anaerobic chamber. Other growth conditions and controls for the dialysis experiments contained empty washed and sterilized tubing and clips. Once removed from the chamber, the headspace of the bottles was exchanged with N_2_:CO_2_ (80%:20%) for 15 min to remove residual H_2_ prior to inoculation. Where indicated, anoxic and 0.22 µm filtered anthraquinone-2,6-disulfonate (AQDS) was added to cultures to a final concentration of 2 mM.

### Quantification of cellular growth, activity, and metal content

Growth of *M. barkeri* Fusaro was monitored via DNA quantification, as a proxy for growth, because the cells strongly attach to FeS_2_ and (Ni,Fe)S_2_ that prohibit accurate cell quantification through microscopy or optical density (OD) approaches. Similar to previously described methods (18), 1 mL of culture was anaerobically and aseptically removed from bottles and centrifuged at 20,000 × *g* for 20 min at 20°C to pellet cells. The supernatant was discarded and the pellets were resuspended in 489 µL of sodium phosphate buffer (MP Biomedicals, Irvine, CA) and 61 µL MT buffer (MP Biomedicals). The cell suspension was subjected to three freeze (−80°C) thaw (70°C) cycles and then transferred to a sterile 2 mL screw-top tube that contained 100 mg of 0.1 mM glass beads (Biospec Products, Bartlesville, OK). The tubes were shaken on a bead beater (Biospec Products) for 40 s and then centrifuged at 14,000 × *g* for 15 min at 20°C to pellet cellular debris, thereby leaving DNA in the supernatant. The DNA concentration was quantified fluorometrically using a Qubit HS dsDNA kit and fluorimeter (Invitrogen, Carlsbad, CA).

To quantify CH_4_, 100 µL of headspace gas from culture bottles was sampled with a N_2_-flushed gas-tight syringe and analyzed via gas chromatograph on a Shimadzu GC-2014 (Shimadzu Scientific Instruments, Columbia, MD) equipped with a 2.0 m HayeSep Q 80/100 column operated at 30°C. A thermal conductivity detector, set at 150°C and 50 mA, was used to measure CH_4_ using ultrahigh purity N_2_ as a carrier gas. A standard curve for CH_4_ was generated using a dilution series prepared from a 100% CH_4_ gas standard (EGAS Depot, Nampa, ID), and this was used to convert the measured peak area to ppm. Total CH_4_ (dissolved and gas phase) was calculated using Henry’s Law. Dissolved HS^-^ in cultures was quantified using the methylene blue assay as described above, and total HS^-^ (dissolved and gas phase) was calculated using Henry’s Law.

Biomass from cultures was analyzed for Ni content by harvesting cultures at the end of growth experiments (day 13) via centrifugation (4,696 × *g* for 20 min at 4°C). The supernatant was removed, and the biomass was resuspended in sterile, anoxic base salts medium with 5 mM nitriloacetic acid to chelate and remove residual metals, including Ni. Biomass was pelleted once more *via* centrifugation (4,696 × *g* for 20 min at 4°C), resuspended in a minimal amount of ultrapure water, and transferred to a pre-weighed, sterile 2 mL screw-top tube. The suspended biomass was dried on a heat block at 70°C overnight and then weighed again to determine biomass dry weight. Next, biomass was digested by dissolving in 0.75 mL of 10% trace metal-free HNO_3_ in a 98°C heat block with periodic vortexing to encourage digestion. Upon complete digestion (~30 h), samples were diluted with 0.75 mL MQ H_2_O and passed through a 0.22 µm PTFE filter prior to analysis on AAS to quantify Ni content, as described above.

### Field emission scanning electron microscopy

*M. barkeri* Fusaro cells were grown as described above with (Ni,Fe)S_2_ as the sole source of Ni, Fe, and S. At mid-log phase, a subsample of cells was collected and subjected to glutaraldehyde fixation and dehydration, as previously described (17). Images were collected using a high-resolution FE-SEM (Supra 55VP, Zeiss, Thornwood, NY) with a primary electron beam energy of 1 keV at different magnifications with technical support from the Imaging and Chemical Analysis Laboratory at Montana State University.

### Bioinformatics screening for nickel uptake protein homologs in methanogens

A previously compiled database of archaeal methanogen and alkanotroph genomes (36) was screened for homologs of NikM (locus tag: MMP1481; gene ID: 2563557431), NikN (MMP1482; 2563557432), NikQ (MMP1483; 2563557433), NikO (MMP1484; 2563557434), and NikR (MMP0020; 2563555938) using protein queries from *Methanococcus maripaludis* S2. An e-value cutoff of 10^−30^ and a query coverage of 60% were specified for identifying protein homologs.

## SUPPLEMENTAL MATERIAL

Supplemental file 1 aem.00991-23-s0001.pdf Supplemental methods, Fig. S1, and Table S1.

Supplemental file 2 aem.00991-23-s0002.xls Table S2.

ASM does not own the copyrights to Supplemental Material that may be linked to, or accessed through, an article. The authors have granted ASM a non-exclusive, world-wide license to publish the Supplemental Material files. Please contact the corresponding author directly for reuse.
