# Supplementary material for: Methanogens acquire and bioaccumulate nickel during reductive dissolution of nickelian pyrite
Source: Appl Environ Microbiol. 2023 Oct 13;89(10):e00991-23. doi: 10.1128/aem.00991-23 (PMC10617489; doi:10.1128/aem.00991-23)
Supplement: Supplemental file 1 — Supplemental methods, Fig. S1, and Table S1. [file aem.00991-23-s0001.pdf]

Methanogens acquire and bioaccumulate nickel during reductive dissolution of nickelian pyrite

Rachel L. Spietz, Devon Payne, and Eric S. Boyd

SUPPLEMENTAL METHODS

Mineral impacts on DNA extraction efficiency.

A single culture of *Methanosarcina barkeri* cells (75 mL volume) was grown under the conditions described in the main text with soluble Fe(II), Ni(II), and sulfide. Once cells reached mid-log phase (day 5), they were split across two gas-tight conical tubes inside of an anaerobic chamber into two and centrifuged at 20,000 x *g* for 20 minutes at 20°C. Inside of the anaerobic chamber, the pelleted cells were resuspended in 5 mL of base salts medium and combined into a single gas-tight conical tube and centrifuged again at the same conditions as above. The washed, pelleted cells were moved back into the anaerobic chamber where the supernatant was decanted and cells were resuspended in 50 mL of sterile base salts medium. The washed cells were distributed across twelve conical tubes by transferring 4 mL into each tube. To three of the tubes, synthetic FeS_2 was added to a final concentration of 2 mM. To the second set of three tubes, $FeCl_2$ was added to a final concentration of 20 μM and Na_2S added to a final concentration of 2 mM. Finally, to all tubes, sterile base salt mediu, was added to bring the volume up to 5 mL. The tubes were incubated at room temperature in the anaerobic chamber for 1 hr to allow cells to interact with the FeS_2 or Fe(II)/HS^-. After incubation, cells were pelleted by centrifugation using parameters described above. DNA was extracted and quantified from each replicate using methods described in the main text.

Supplemental Figure and Table

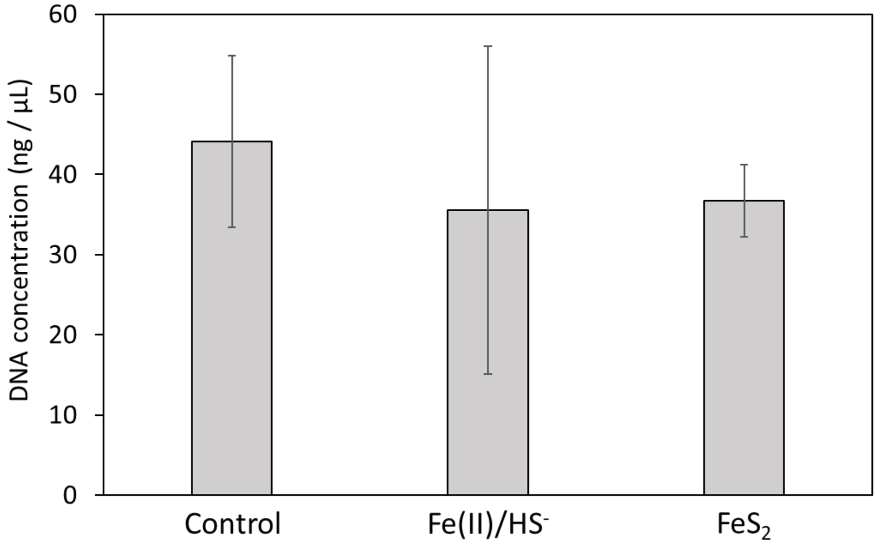

FIGURE S1. The DNA yield from *Methanosarcina barkeri* cells during extractions conducted in the absence (control) or in the presence of aqueous iron and sulfide (Fe(II)/HS^-) or synthetic pyrite (FeS_2).

TABLE S1. Cellular nickel content of *Methanosarcina barkeri* Fusaro normalized to either DNA yield or to dry weight biomass.

[1] data represents the mean and standard deviation of the mean (n=3)
HS^-, sulfide
Fe(II), soluble iron

	Ni content normalized to DNA yield (ng Ni/µg DNA) [1]	Ni content normalized to dry weight biomass (ng Ni/mg dry weight) [1]
**Ni-deplete (HS^-/Fe(II))**	1.69 ± 0.24	6.40 ± 1.08
**Ni-replete (HS^-/Fe(II)/Ni(II))**	4.22 ± 0.33	35.05 ± 4.98
**Ni-leached (Seq. $(Ni,Fe)S_2$/HS^-/Fe(II))**	2.05 ± 0.54	10.02 ± 1.99
**Ni-mineral (Seq. $(Ni,Fe)S_2$ + AQDS/HS^-/Fe(II))**	15.70 ± 2.50	163.19 ± 12.81

Ni(II), soluble nickel
Seq. $(Ni,Fe)S_2$, sequestered nickelian pyrite in dialysis tubing
AQDS, anthraquinone-2,6-disulfonate
